# Fitting into the collective: a cultural regulatory fit perspective on international student adaptation in China

**DOI:** 10.3389/fpsyg.2025.1659368

**Published:** 2025-08-27

**Authors:** Jiayu Li, Ting Zhang

**Affiliations:** ^1^Zhejiang Normal University, Jinhua, China; ^2^Zhejiang Industry Polytechnic College, Shaoxing, China

**Keywords:** cultural regulatory fit (CRF), subjective wellbeing (SWB), cultural adaptation (CA), identity strain (IS), individualistic value orientation (IVO), cross-cultural

## Abstract

Against the backdrop of China's growing international student population, enhancing their subjective wellbeing has become a pressing issue in university management. Drawing on cultural regulatory fit theory, This study investigates the influence mechanism of cultural regulatory fit on the subjective wellbeing of international students in China based on data collected at three time points. The final valid sample size after matching across all three stages is *N* = 327. A dual mediation model was proposed, incorporating cultural adaptation and identity strain, with individualistic value orientation as a moderator. Results revealed that: (1) cultural regulatory fit promotes adaptation, reduces identity strain, and enhances wellbeing; (2) both mediators play significant roles in this process; and (3) individualistic orientation did not significantly moderate the main pathway, but multi-group analyses confirmed the model's cross-cultural applicability across Asian and non-Asian groups. This study contributes a novel perspective on the dynamic cognitive-emotional mechanisms of adaptation and offers practical insights for psychological support strategies in higher education.

## 1 Introduction

With the continuous advancement of China's internationalization strategy for higher education, the number of international students in China is increasing, and China is gradually becoming one of the most attractive destinations for studying abroad among non-English speaking countries. According to the Ministry of Education (2023), despite the short-term impact of the COVID-19 pandemic, the number of international students at Chinese universities is on the rise, forming a diverse structure dominated by Asia, Africa, and countries along the Belt and Road. At present, a growing number of studies have noticed that issues such as cultural shock, identity imbalance, and adaptation pressure faced by international students during cross-cultural migration are not only marginal topics of academic adaptation, but also important variables affecting their overall wellbeing and mental health (Takyi Mensah et al., 2024). Especially in China's highly collectivist cultural context, international students who are dominated by individualistic values often face problems such as value cognition conflicts, social style differences, and lack of social belonging (Chu and Zhu, 2023), which makes exploring the deep mechanisms between cultural value orientation and psychological adjustment an urgent theoretical issue to respond to.

In existing studies, individualism and collectivism have long been used as core dimensions of cross-cultural psychological research to explain attitudinal differences in cultural adaptation (Maheshkar and Sonwalkar, 2023). In recent years, as cultural psychology has shifted from “comparison of cultural differences” to “cultural dynamic fit”, researchers have gradually recognized that cultural adaptation is not a linear process of cultural transfer, but rather the result of continuous interaction and adjustment between the individual and the context (Ayoko et al., 2022). For example, Alshammari et al. (2023) found that international students are more likely to achieve identity integration and psychological belonging when facing the collectivist values of the host country if they can actively understand and identify with its normative expectations. However, in contrast, some studies have pointed out that when an individual's cultural values are inconsistent with their environmental value orientation, they are prone to identity conflicts and cultural disembedding, thereby affecting their overall wellbeing and cultural identity level (Peiren, 2023). This suggests that cultural values do not have a one-way influence, but rather require “value alignment” and meaning construction through the individual regulatory system (Stray et al., 2024).

Although the above research expands the understanding of the impact of cultural value dimensions on adaptation outcomes, there are still three deficiencies in current mainstream research. First, most studies treat cultural values as static background variables and ignore the active moderating ability that individuals play in cultural adaptation. Especially in contexts where there is a significant cultural gap, merely emphasizing “cultural distance” or “degree of cultural identity” is not sufficient to explain the differences in adaptation outcomes (Cesario et al., 2004; Nakkawita and Higgins, 2024; Vignoles et al., 2016). Secondly, the existing literature generally neglects the matching mechanism between an individual's regulatory focus and cultural context. Regulatory Fit Theory (RFT) posits that individuals experience enhanced motivational strength, cognitive fluency, and psychological engagement when their regulatory focus (promotion or prevention) aligns with the contextual incentive structure (Nakkawita and Higgins, 2024). Although this theory has been extensively applied in consumer behavior and organizational psychology, its application in the context of international students‘ cross-cultural adaptation remains limited. Recent studies have emphasized the importance of examining how motivational systems embedded in cultural contexts influence adaptation processes (Sheng et al., 2022; Bui et al., 2021). However, few empirical studies have directly tested whether a match between students' regulatory focus and host culture incentive cues enhances their psychological wellbeing and adaptation. Thirdly, although some studies have noted the dual-path possibility of adaptation (such as the coexistence of “adaptation and suppression”), they lack modeling of the psychological mechanisms in the process of cultural fit. In particular, it remains unclear whether cultural regulatory fit enhances wellbeing by promoting cultural identity or by reducing identity strain, two pathways that have theoretical relevance but insufficient empirical validation (Bierwiaczonek and Kunst, 2021; du Toit et al., 2022).

Based on the above research gaps and theoretical challenges, this paper proposes CRF as the core explanatory mechanism, constructs a matching model that integrates an individual's regulatory focus with perceived collectivism, and systematically explores how international students in China can achieve CA, IS, and happiness enhancement through the regulatory fit mechanism. Unlike the traditional view of cultural values as external constraints, this article emphasizes that the “collectivist incentive framework” in the cultural environment is not a single obstacle, but may be positively internalized under a specific regulatory structure and transformed into psychological resources for individual behavioral adaptation. By introducing the accommodative fit path, this paper breaks the linear understanding of “cultural adaptation is cultural compliance” and constructs a three-stage process model of “cultural orientation—accommodative structure—adaptive outcome”, which not only provides a new mechanical-level explanatory framework for cultural psychology, it also provides empirical references for Chinese universities to achieve differentiated psychological support and cultural guidance in the increasingly diverse management practices of international students.

## 2 Theoretical developments and research hypotheses

### 2.1 The relationship between CRF and CA

CA is not merely the process of shaping individual behavior by external cultural norms, but rather an interactive mechanism of bidirectional regulation and meaning construction between the individual and the cultural environment (Volet and Jones, 2012). Especially in a highly collectivist cultural context, international students in China need to constantly adjust their behavioral strategies and motivational patterns to adapt to social situations where there is a significant cultural gap. The RFT provides an important perspective for analyzing this adaptation process. This theory holds that when an individual's regulatory focus is consistent with the incentive methods emphasized in the situation they are in, stronger cognitive fluency, a sense of goal clarity and behavioral efficacy will be generated, thereby enhancing the individual's adaptation to the environment and perception of meaning (Higgins, 2005; Cloos et al., 2023).

In the context of CA, CRF can be defined as the degree of match between an individual's regulatory focus and the perceived cultural value orientation of the host country. For international students, a positive sense of fit may be formed if there is a high cognitive consistency between their motivational orientation (such as the promoting pursuit of personal growth and self-improvement) and the normative requirements emphasized in Chinese culture, such as collective responsibility and social harmony. This fit helps to reduce cultural discomfort, relieve psychological stress, and thereby improve the quality of CA (Sangraula et al., 2021). Conversely, when there is a mismatch between the modulation and cultural expectations, that is, when individuals tend to express themselves autonomically and make personal decisions, but the cultural environment emphasizes conformity, respect for authority and collective coordination, the degree of fit is reduced and the adaptation process may be interrupted or distorted.

Studies have shown that in situations with a high level of regulatory fit, individuals tend to exhibit a higher willingness to participate, a stronger sense of cultural identity, and more positive behavioral feedback (Cesario et al., 2008). For instance, An and Chiang (2015) found that in the Chinese cultural context, the degree of matching between regulatory focus and cultural identity can effectively predict the CA degree of international students. Therefore, this paper puts forward the following hypothesis:

H1: The higher the degree of CRF, the higher the CA level of international students in China.

### 2.2 The relationship between CRF and SWB

In the process of CA, wellbeing is not only the result of external cultural integration, but also an important psychological indicator of whether positive interaction can be achieved between the individual and the environment (Chen et al., 2014). In recent years, research on SWB has gradually shifted from static measurement of emotional states and life satisfaction to exploration of the intrinsic regulatory mechanisms of individuals in social and cultural contexts (Iacus and Porro, 2021). For international students in China, their wellbeing depends not only on the availability of external resources, but more deeply on whether they can align and resonate with mainstream cultural values through the adjustment of motivational systems in an unfamiliar cultural context (Di et al., 2022). The RFT provides the key psychological mechanism for this process. The theory suggests that when an individual's goal-pursuing style aligns with the behavioral tendencies supported by the environment, a so-called “accommodative fit” is formed. This fit not only enhances task engagement and goal persistence but also stimulates value consistency and sense of meaning in subjective experience, leading to a higher level of emotional satisfaction and psychological efficacy (Higgins, 2005; Cesario et al., 2008).

In a cross-cultural context, this mechanism of adjustment and fit is particularly important. For international students in a collectivist cultural context, if they themselves possess a promoting or preventive regulatory focus and can identify and smoothly adapt to the behavioral incentive methods in Chinese culture that emphasize social responsibility, group belonging, and compliance with norms, they will be more likely to achieve a consistent experience of “cognition—behavior—culture”. This sense of fit not only enhances their adaptability confidence and cultural identity, but also provides a source of psychological energy for them in aspects such as learning, interpersonal communication and life, effectively improving their SWB level (Bakhti et al., 2022). Conversely, if there is a tension between an individual's motivational style and cultural orientation, such as an individual valuing personal expression and independent decision-making, but the cultural context encourages relationship compliance and collective coordination, there will be a “regulatory misalignment” in their psychological energy system, which leads to confusion, self-denial and value conflict, thereby weakening their wellbeing (Noels et al., 2020).

Accommodative fit also enhances the structural stability of wellbeing by stimulating internal consistency and meaning construction. According to self-determination theory, an individual's wellbeing is based on the satisfaction of three psychological needs: autonomy, relationality, and competence (Autin et al., 2022). When the degree of cultural fit is high, international students are more likely to feel the rationality of their own choices in behavior, gain a sense of security in social connection in relationships, and experience consistency with mainstream cultural goals in cognition. This structural psychological consistency is precisely the core source of the formation of “eudaimonic wellbeing” (Ludwigs et al., 2019). The “motivational fit” brought about by moderating fit makes it easier for individuals to interpret the CA process as an active growth opportunity rather than a passive compromise behavior, which not only reduces cultural stress but also stimulates an intrinsic sense of meaning, further enhancing their overall positive evaluation of the study abroad experience.

Therefore, this paper puts forward the following hypothesis:

H2: The higher the degree of CRF, the stronger the SWB of international students in China.

### 2.3 The mediating role of CA

Regulatory fit, as a psychological state in which subjective cognition matches situational factors, not only directly enhances an individual's positive experience, but also indirectly affects psychological wellbeing through specific behavioral and cognitive mechanisms. CA, as a key variable in the process of cross-cultural migration, is widely regarded as an important intermediary bridge connecting the external cultural environment with the individual's internal psychological experience (Ward and Kennedy, 1999; Boski et al., 2025). For international students in China, CA is not only reflected in behavioral aspects such as language, lifestyle, and classroom interaction, but also in the recognition of the Chinese cultural value system and the understanding and acceptance of collectivist social norms. Existing studies have shown that international students with a high level of CA tend to have a stronger sense of belonging, lower cultural stress, and more positive emotional responses. These factors jointly enhance their SWB level (Alshammari et al., 2023).

The impact of CRF on happiness largely depends on whether it can facilitate an effective CA process. When an individual's regulatory focus (such as the promoting type tending to proactively engage with and learn about new cultures, and the preventive type placing more emphasis on risk control and relationship maintenance) aligns with the value orientation of the eastern culture (such as emphasizing obedience, harmonious relationships, and collective goals), the individual is more likely to understand and accept the behavioral rules and communication logic within that culture. Thus reducing cultural frictions and misunderstandings (Noels et al., 2020). For example, facility-oriented individuals are more likely to actively participate in local social networks and group activities in Chinese culture, thereby establishing an effective social support system; Preventive individuals will also be more willing to maintain their relationship with the local community if they perceive that the eastern culture also values norms and responsibility, thus forming a stable cultural link (Cloos et al., 2023). Under these conditions, CRF can enhance an individual's level of CA by improving their understanding and adaptability to the cultural environment.

CA as a mediating mechanism, connects the two dimensions of “cognitive fit,” and “emotional wellbeing”. According to the dual-process model of acculturation proposed by Ward and Kennedy (1999), CA includes two levels: affective adaptation and behavioral adaptation. Accommodative fit contributes to the simultaneous advancement of both: the former is manifested as emotional acceptance and identification with Chinese culture, and the latter is manifested as the gradual formation of effective interaction in daily life and academic communication. When these two adaptations are established, the individual not only reduces cultural stress but also experiences positive meaning and self-worth in a cross-cultural environment, thereby achieving a higher level of wellbeing (Cui and Zhang, 2024).

In contrast, if the accommodative fit is low, individuals cannot achieve a match at the value level, and their adaptive behaviors are often restricted, making them prone to cultural resistance, social avoidance and cognitive conflicts. This not only hinders the development of their cultural learning path, but also causes individuals to have a negative assessment of the cultural environment, thereby suppressing the formation of happiness. Therefore, CA as an important mediating path between regulating fit and wellbeing, is not only an external indicator at the behavioral level, but also an important result of the integration of individual psychological regulation and cultural cognition.

Based on the above theory and empirical basis, the following hypotheses are proposed:

H3a: CRF positively predicts CA among international students in China.
H3b: CA positively predicts SWB among international students in China.
H3c: CA mediates the relationship between CRF and SWB.

### 2.4 The mediating role of IS

Although CRF can help enhance an individual's level of CA and wellbeing, this process is not a single positive path. For cross-cultural contexts with significant differences in values, the adaptation process is often accompanied by complex identity reconstruction challenges (Berry, 2005). Especially in highly collectivist cultural Settings, international students in China not only need to understand and accept specific behavioral norms and social expectations, but also often face the tension between identity consistency and cultural loyalty. When individuals fail to achieve effective coordination between their own cultural identity and the cultural expectations of the host country, what is called “identity strain” or “cultural identity conflict” occurs, manifested as phenomena such as ambiguous cultural roles, disharmony of values, limited self-expression and inconsistency between internal and external behaviors (Maertz et al., 2016; Nguyen and Benet-Martínez, 2013).

The emergence of IS often stems from the lack of regulatory fit. When an individual's habitual motivational style conflicts with the motivational structure of the cultural environment, such as the individual's pursuit of autonomy, self-expression and independence, while the collectivist culture emphasizes obedience to authority, collective interests first and social harmony, the individual has to sacrifice some self-expression to adapt to the environment. In this process, if the individual is unable to achieve value integration through the internal construction mechanism, the cultural adjustment process will turn to “self-repression” and “apparent compliance”, resulting in long-term psychological consumption and value fragmentation (Stray et al., 2024). This sense of inconsistency constitutes the core source of IS and is also regarded as one of the important precursors of failed cross-cultural adaptation.

IS is not only an emotional response to cultural conflicts, but also has persistent structural psychological consequences. Studies have shown that identity conflicts can significantly weaken an individual's sense of belonging, self-esteem and wellbeing, and may even induce psychological symptoms such as anxiety and depression (Tafarodi et al., 2012; Rahim et al., 2021). In the group of international students in China, when cultural regulation fails to bring about identity integration, individuals may exhibit formal integration in behavior and remain in a “psychological marginal state” emotionally, making it difficult to truly achieve cultural resonance and emotional stability. Compared with the short-term distress caused by insufficient behavioral adaptation, the erosion of happiness by IS is more concealed and persistent. Its negative impact often accumulates over a long period of time and is difficult to solve through short-term intervention.

Therefore, this paper proposes that although CRF can directly enhance wellbeing to a certain extent, its effect may also exert an indirect influence through the negative psychological mechanism of IS. When the fit is low, the more difficult it is for an individual to internalize the culture, the more likely they are to feel the inconsistency between their identity and the cultural environment, which in turn causes psychological distress and inhibits the generation of wellbeing.

Based on the above theoretical reasoning and empirical basis, this paper proposes the following hypothesis:

H4a: CRF negatively predicts IS among international students in China.
H4b: IS negatively predicts SWB among international students in China.
H4c: IS mediates the relationship between CRF and SWB.

### 2.5 The moderating role of IVO

The positive effect of CRF on SWB does not have the same intensity in all populations, and its effect may be regulated by the internal cultural value orientation of individuals. Cultural values, as a deep psychological structure in the process of individual socialization, determine their cognitive assessment criteria and behavioral preferences in unfamiliar cultural Settings (Hofstede, 2011). In cross-cultural psychological research, individualistic or collectivist value orientations are widely used to predict an individual's sensitivity to cultural differences, the choice of adaptation strategies, and the degree of willingness to internalize culture (Chirkov et al., 2003). For international students in China, the IVO reflects their preference for autonomy, self-expression, and personal goals, and this orientation may significantly influence their situational assessment and psychological response to Chinese collectivist culture.

International students with a higher individualistic orientation are more likely to have a mismatch between their motivational systems and environmental expectations when they are in a collectivism-dominated cultural environment. This cognitive difference intensifies their perceived sensitivity to cultural differences, making them more dependent on internal fit judgments for cultural assessment and emotional regulation (Wu and Zhao, 2021). Against this backdrop, once an individual can align their regulatory focus with cultural incentive methods, the sense of regulatory fit they achieve will become even more significant, as it may serve as their “value breathing hole under cultural pressure”, thereby generating significant positive returns at the emotional experience level. That is to say, among individuals with a strong individualistic orientation, the positive predictive path of CRF for wellbeing will be more prominent. This mechanism can be seen as an adaptive buffering effect under the condition of “culture-motivation-value inconsistency”, that is, the IS mechanism provided by moderating fit shows stronger psychological compensatory power in a context of high cultural tension.

On the contrary, for international students with a strong collectivist value orientation, there is a higher natural consistency between their cultural behavior and Chinese social norms. In this context, accommodative fit still plays a role, but because the value structure itself is more compatible, individuals are less sensitive to fit, and their level of wellbeing is more likely to be influenced by direct social connections, collective belonging, and familiarity with the situation. Therefore, the marginal effect of moderating fit as a mediating mechanism is relatively low in such groups and is no longer the core path affecting wellbeing.

In addition, individualistic values not only influence the choice of adaptation strategies, but also the individual's path of meaning construction in the cultural environment, especially their role in motivation consistency and the formation of a sense of belonging cannot be ignored (Sagiv and Schwartz, 2022). Therefore, the IVO not only forms the background basis for the formation of cultural fit, but also determines whether an individual can gain psychological benefits from it after the formation of fit.

Based on the above reasoning, the following hypothesis is proposed:

H5a: IVO moderates the effect of CRF on CA.
H5b: IVO moderates the effect of CRF on IS.
H5c: IVO moderates the direct effect of CRF on SWB.

**Figure 1:**
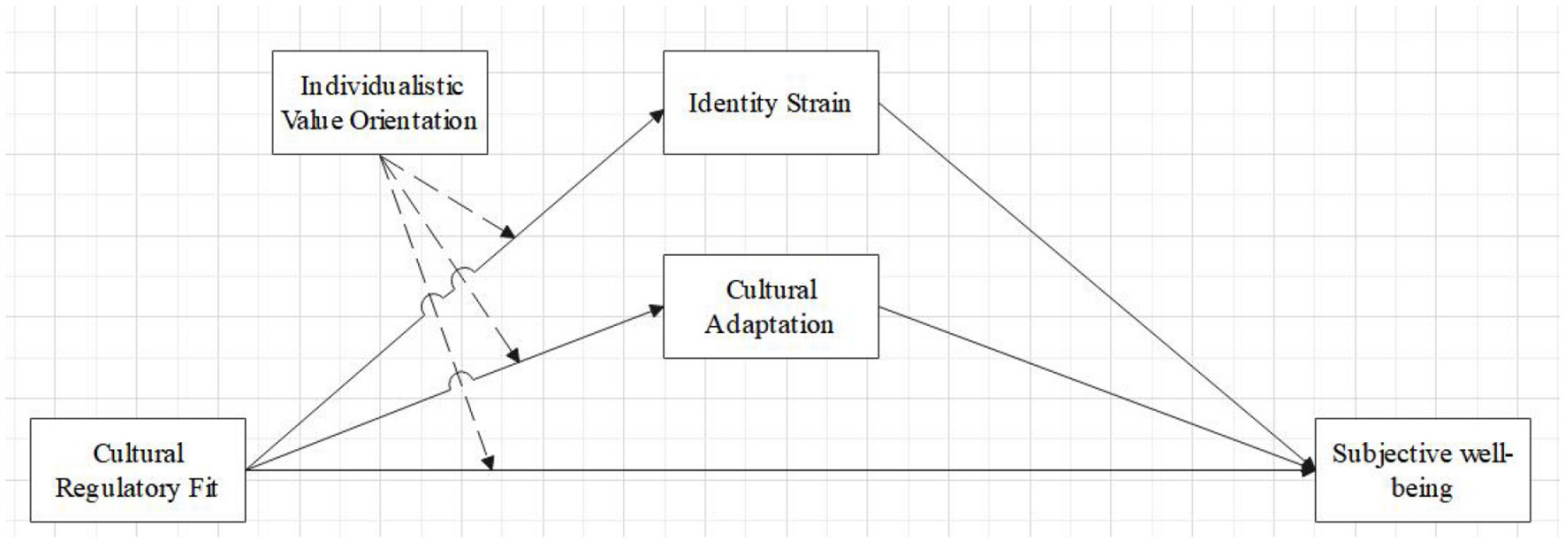
Research framework.

The research framework of this study is shown in Figure 1.

## 3 Methodology

### 3.1 Research subjects and data collection

**Figure 2:**
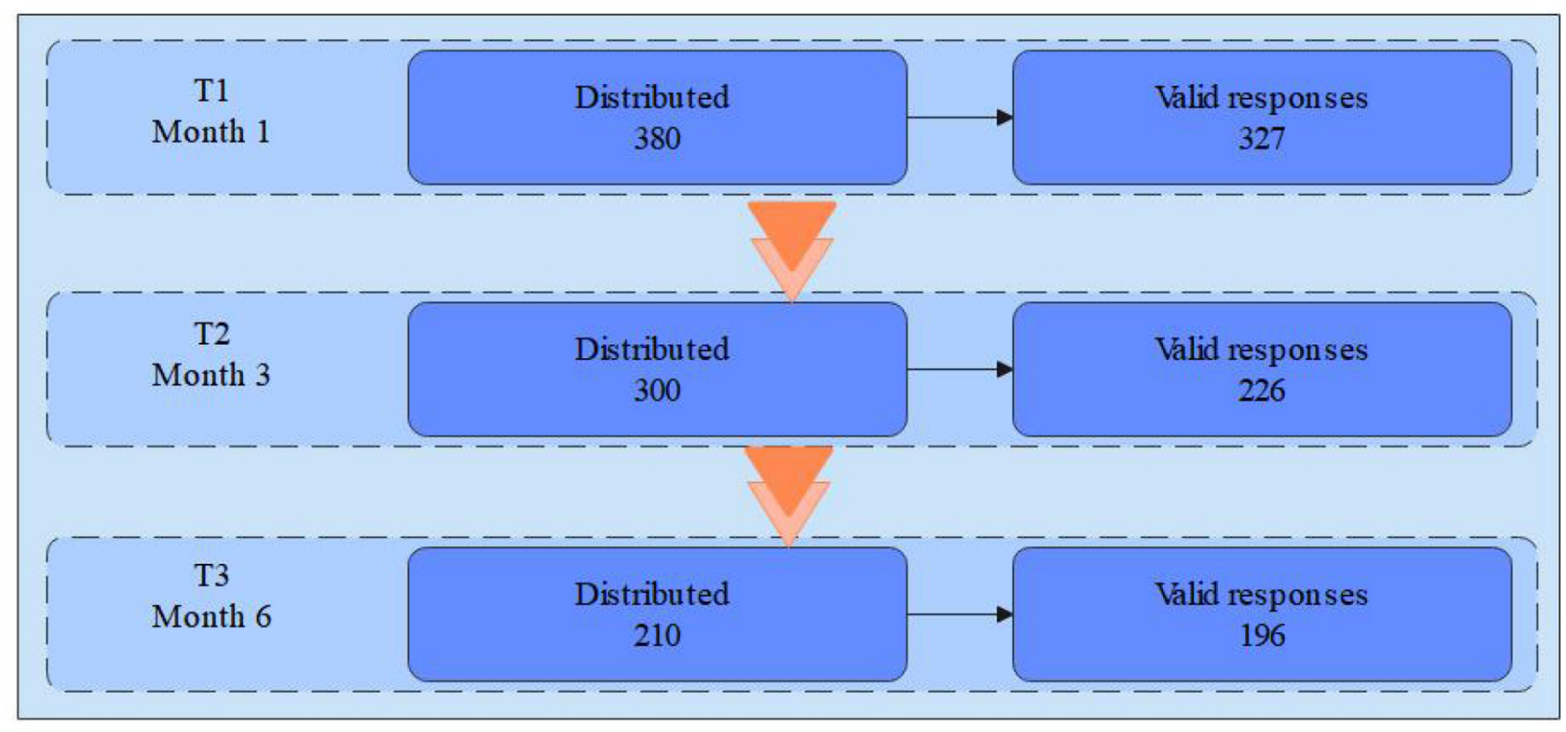
Measurement process.

This study employed a multi-stage follow-up questionnaire design to enhance the causal inference ability of the model and capture the temporal evolution of psychological variables in international students during adaptation. The entire study period lasted for 6 months, with three measurement time points (T1, T2, T3) set, each about 2 months apart, to measure different core variables. The specific arrangements are as follows:

Phase T1 (Month 1): 380 questionnaires were distributed and 327 valid responses were collected. Study 1 used T1 data only to examine the relationship between CRF, IVO, and CA;

Phase T2 (Month 3): Study 2 focused on testing a dual mediation model and moderation effects based on the paired data from T1 and T2 (*N* = 226). This included the mediating variables CA and IS, which were measured at T2, as well as the moderator variable IVO. The measurement samples for three time periods are shown in Figure 2.

Phase T3 (Month 6): Study 3 used the data collected at T3, in which 210 questionnaires were distributed and 196 valid responses were obtained. This dataset served as a delayed measurement of SWB to conduct robustness tests, verify temporal stability, and examine cross-cultural invariance through multi-group comparisons.

Each stage questionnaire was sent by the research team to the participants reserved email via an online questionnaire platform, such as Wenjuanxing, with a unique anonymous tracking code set for matching and data integration. To increase tracking rates and sample retention rates, the research team provided small rewards to respondents who completed all three measurements and continued communication and reminders through the Office of International Students. A total of 336 valid samples with complete data for all three phases were obtained, and the overall sample loss rate was controlled within 20%, meeting the criteria for follow-up studies. This design not only allows us to separate anagen, mediator and outcome variables in the time dimension, but also effectively alleviates the common method bias problem commonly found in cross-sectional designs.

Before the formal implementation of the questionnaire, the research team invited five experts in cross-cultural psychology to review the content of the questionnaire and conducted a pre-test on 20 informal samples to ensure the comprehensibility and cultural fit of the scale content. Before the questionnaire survey, the researchers explained the purpose of the study, the principle of anonymity and the use of the data to all participants and obtained their informed consent. The entire research process followed the Ethical Guidelines for Psychological Research to ensure that participants' rights were not violated.

### 3.2 Measures

#### a. Variable measurements

Five core variables were included in this study. They are respectively CRF, CA, and IS, SWB and IVO. All variables were measured by structured questionnaires, with items scored using the five-point Likert scale (1 = strongly disagree, 5 = strongly agree) to ensure cultural fit and operational consistency in understanding and scoring among respondents.

CRF, as the key predictor variable of this study, consists of six items and comprehensively measures the degree of subjective matching between an individual's own regulatory style and the value orientation of the host culture. The scale references a localized revision of the cultural context adaptation by Pan (2017), emphasizing cognitive experiences such as “I feel that my behavioral style matches the value orientation of Chinese culture.”

CA, as one of the mediating variables, consists of six items and measures the degree of adaptation of international students to the Chinese cultural environment at both the emotional and behavioral levels. The scale is adapted to the two-dimensional cultural adaptation framework proposed by Ward and Kennedy (1999) and is used to assess the overall adaptation status in the process of cultural environment integration.

IS consists of six items, measuring an individual's perception of psychological tension such as role conflicts, value contradictions, and identity confusion during the process of cultural identity integration. This scale refers to the cultural identity conflict framework developed by Ward et al. (2011) and is applicable to capturing common internal psychological frictions in cross-cultural environments.

SWB, as the outcome variable of this study, consists of five items, selected from the core dimensions of the PERMA model proposed by Seligman (2011), including positive emotions, sense of meaning, relationship quality and achievement experience, etc. The scale emphasizes the psychological dimension of quality wellbeing and is suitable for assessing long-term psychological wellbeing after cultural adaptation.

IVO was used as a moderating variable, consisting of six items, and referred to Sivadas et al. (2008) for the division of horizontal individualism and vertical individualism dimensions to measure the degree of value recognition of self-expression, independent judgment, and social evaluation by respondents. The scale has been widely used to identify the cultural tendencies of individualism in cross-cultural contexts.

#### b. Common method bias

Given that all the variables in this study were collected from the same source through self-administered questionnaires, there is a risk that Common Method Variance (CMV) may interfere with the relationships among the variables. To detect and control the CMV problem, Harman's Single-Factor Test and Unmeasured Latent Method Construct (ULMC) were used for verification in this study.

Harman's Single-Factor Test is one of the most commonly used statistical techniques for assessing Common Method Bias (CMB). In this test, all measurement items are subjected to an Exploratory Factor Analysis (EFA) without rotation, and if a single factor emerges or one general factor accounts for more than 50% of the variance, it suggests the presence of common method variance (Podsakoff et al., 2003). In the Harman single-factor test, all measurement items were included in the unrotated exploratory factor analysis. The analysis results showed that the total variance explained by the first principal factor was 25.264%, well below the 50% warning line, indicating that the single factor did not dominate most of the variation and initially ruled out the risk of significant common method bias (Podsakoff et al., 2003; Fuller et al., 2016).

Furthermore, to enhance the robustness of CMV detection, this study adopted the ULMC method. The ULMC is a more rigorous confirmatory method for evaluating CMB. In this approach, a common latent factor is added to a Confirmatory Factor Analysis (CFA) model, and all observed variables are allowed to load onto it. If the method factor does not explain substantial variance in the observed variables, common method bias is unlikely to be a major concern (Podsakoff et al., 2012). To evaluate model fit, several indices were used, including χ^2^/df, CFI, RMSEA, and SRMR. According to standard criteria, CFI ≥ 0.90 indicates acceptable fit and ≥ 0.95 indicates good fit; RMSEA < 0.08 is acceptable and < 0.06 is considered good; SRMR < 0.08 is acceptable (Hu and Bentler, 1999; Kline, 2023). To test for CMV a latent method factor was added to the CFA model. In the baseline model, the fit was good (χ^2^/df = 2.347, CFI = 0.948, RMSEA = 0.052, SRMR = 0.047). The extended model with the method factor showed only slight improvement (χ^2^/df = 2.276, CFI = 0.954, RMSEA = 0.049, SRMR = 0.045), and the change in CFI (ΔCFI = 0.006) did not exceed the 0.01 threshold (Williams et al., 1989). Together with Harman's single-factor test, these results suggest that CMV is not a major concern in this study.

#### c. Measurement model

To verify the reliability and validity of the measurement tools, this study employed CFA within a Structural Equation Modeling (SEM) framework. As shown in Table 1, all standardized factor loadings exceeded the recommended threshold of 0.70 (ranging from 0.721 to 0.914), demonstrating strong indicator reliability (Hair, 2014). The Cronbach's α coefficients for all latent constructs were above 0.80 (0.821 to 0.928), and the Composite Reliability (CR) values were all greater than 0.88, indicating high internal consistency and construct reliability (Hair, 2014). Additionally, the average variance extracted (AVE) values ranged from 0.553 to 0.803, surpassing the 0.50 threshold and supporting convergent validity (Fornell and Larcker, 1981).

**Table 1:** CFA analysis.

**Construct**	**Item label**	**Standardized loading**	**Cronbach's α**	**C.R**	**AVE**
CRF	CRF1	0.85	0.901	0.909	0.672
	CRF2	0.833			
	CRF3	0.768			
	CRF4	0.827			
	CRF5	0.727			
	CRF6	0.836			
CA	CA1	0.795	0.87	0.834	0.692
	CA2	0.757			
	CA3	0.815			
	CA4	0.813			
	CA5	0.744			
	CA6	0.715			
IS	IS1	0.779	0.861	0.863	0.677
	IS2	0.801			
	IS3	0.85			
	IS4	0.859			
	IS5	0.795			
	IS6	0.831			
IVO	IVO1	0.713	0.906	0.861	0.604
	IVO2	0.826			
	IVO3	0.844			
	IVO4	0.774			
	IVO5	0.782			
	IVO6	0.846			
SWB	SWB1	0.78	0.878	0.877	0.718
	SWB2	0.829			
	SWB3	0.743			
	SWB4	0.769			
	SWB5	0.811			

Further, this study evaluated the discriminant validity between latent variables based on the Fornell-Larcker discriminant validity test. As shown in Table 2, the AVE square root (diagonal) values of all latent variables were greater than their correlation coefficients with other latent variables (non-diagonal), indicating good discriminant validity among constructs. For example, the AVE square root of CRF is 0.824, significantly higher than its correlations with CA (0.611), IS (0.482), SWB (0.519), and IVO (0.493), and comparisons among other variables also conform to this criterion.

**Table 2:** Discriminant validity.

**Construct**	**CRF**	**CA**	**IS**	**IVO**	**SWB**	**VIF**
CRF	0.825	0.408	0.615	0.645	0.541	1.78
CA	0.568	0.772	0.376	0.307	0.57	1.94
IS	0.535	0.464	0.776	0.599	0.602	3.12
IVO	0.408	0.63	0.363	0.802	0.532	1.95
SWB	0.308	0.513	0.429	0.591	0.815	2.95

In addition, to rule out potential multicollinearity issues, the study also calculated the Variance Inflation Factor (VIF) for each latent variable. As shown in Table 2, the VIF values of all variables were between 1.780 and 3.120, well below the general threshold of 10 for judging multicollinearity, indicating that there was no serious risk of multicollinearity in this study (Hair, 2014). Therefore, the combined results of factor loading, reliability index, aggregated validity and discriminant validity confirm that the measurement model of this study has good measurement quality.

### 3.3 Data analysis methods

To comprehensively test the proposed mediating effect and moderating effect paths, in the data analysis stage of this study, SPSS 26.0 combined with PROCESS was used for multi-step path analysis. First, the raw data were preprocessed using SPSS, including missing value processing, sample matching, norm statistical description and variable distribution test, and invalid samples that did not complete the three-stage questionnaire were excluded to ensure the completeness and temporal consistency of the analyzed samples.

In terms of variable relationship testing, multiple models in the PROCESS were adopted to verify how cultural regulatory fit affects subjective wellbeing through cultural adaptation and identity strain, and to investigate the possible moderating effect of individualistic value orientation in this process. Specifically, Model 4 was used to test the dual mediating path of CRF (X) → CA/IS (M) → SWB (Y); Then Model 1 and Model 14 were used to test the moderating effect of IVO (W) on the X → Y direct path and the X → M path respectively; For further verification of the significance of the moderating mediating effect, Model 58 is used to assess the moderating effect of the moderating variable on the entire indirect path, that is, whether there is a “conditional indirect effect”.

All path analyses were tested for significance using bias-corrected bootstrapping, with the number of repeated samples set at 5,000 times and 95% bias-corrected confidence intervals constructed. If the confidence interval does not contain zero, the path effect is considered statistically significant (Hayes, 2017). In addition, to enhance the explanatory power of the model, demographic variables such as gender, age, and length of residence in China were controlled for inclusion as covariates in the model.

The overall analysis path design helps to deeply reveal how CRF affects the wellbeing of international students through multiple psychological mechanisms, and further clarify the regulatory role of IVO in the process of cross-cultural adaptation, providing rigorous empirical support for understanding the dynamic relationship between CA and SWB.

## 4 Empirical analysis

### 4.1 Study1 empirical test

**Objective:** To verify the impact of CRF on CA, And to explore the moderating effect of IVO, this study is based on 327 valid samples from stage T1 and uses moderating regression analysis for the test.

**Participants:** In Study 1 (T1 stage), a total of 380 international students from multiple universities in China were invited to participate, and 327 valid responses were collected. Participants were recruited using stratified sampling to ensure diversity across geographic and institutional contexts. Specifically, 56.8% of the participants were enrolled in universities located in eastern China, 23.4% in central China, and 19.8% in western China. In terms of academic level, 68.9% of participants were undergraduate students, 24.5% were master's students, and 6.6% were doctoral students. Regarding gender distribution, 48.3% identified as male, and 51.7% as female. The majority of respondents were aged 18–25 years old (58.3%), followed by those aged 26–35 (29.7%). Participants came from a broad range of cultural backgrounds, including Asia (52.6%), Africa (21.8%), Europe (15.5%), and other regions such as South America and Oceania (10.1%). Their academic disciplines spanned science and engineering, business, humanities, and other fields relevant to cross-cultural research. The duration of participants' stay in China ranged from 3 to 48 months, with a mean of 17.4 months (SD = 8.7). All participants demonstrated sufficient proficiency in English or Chinese to understand and complete the questionnaire.

**Results:** As shown in Table 3, CRF has a significant positive predictive effect on CA (β = 0.584, *p* = 0.005), supporting hypothesis H1, indicating that when the behavioral patterns of international students in China are more in line with the orientation of Chinese culture, their level of CA significantly improves. The influence of IVO itself on CA is not significant (β = 0.293, *p* = 0.169), and the interaction term of its fit with CRF does not reach a significant level (β = −0.027, *p* = 0.562), which does not support the moderating effect hypothesis H2. This indicates that in the sample of this study, the degree of individual recognition of independence and autonomous value does not significantly modulate the path of CRF on CA.

**Table 3:** Results of regression analysis in study1.

**Variables**	**β value**	**SE**	***t* value**	***p* value**	**Lower 95% CI**	**Upper 95% CI**
Constant term	−0.189	0.931	−0.203	0.840	−2.020	1.643
CRF	0.584^**^	0.205	2.853	0.005	0.181	0.987
IVO	0.293	0.212	1.379	0.169	−0.125	0.710
CRF × IVO	−0.027	0.047	−0.580	0.562	−0.119	0.065
*R* ^2^	0.270					
*F*	37.529					

** *p* < 0.01.

The overall model fits well with a coefficient of determination *R*^2^ = 0.270, indicating that the model is significant (*F* = 36.529, *p* < 0.001), suggesting that the independent variables have some explanatory power for CA. However, the small increment of *R*^2^ in the interaction term (Δ*R*^2^ = 0.001) further validates the insignificance of the moderating effect. Combined with the above results, Study 1 preliminarily verified the importance of CRF as a anvariate variable. However, the moderating role of individualistic values has not yet been empirically supported, and it may be necessary to further investigate its performance in subsequent psychological mechanisms.

### 4.2 Study2 empirical test

**Objective:** Study 2 aims to be based on paired samples from phases T1 and T2, To further verify how CRF indirectly affects the SWB of international students in China through CA and IS, and examine the moderating effect of IVO on the relationship between CRF and SWB.

**Participants:** In Study 2 (T2 stage), we adopted a multi-stage paired tracking design to follow up with participants from the initial Study 1 sample. Out of the 300 participants who consented to be re-contacted, we successfully tracked and obtained 226 valid responses, yielding a tracking retention rate of 75.3%. The follow-up sample preserved strong demographic and cultural diversity. Regarding gender, 46.0% of respondents were male, and 54.0% were female. The age distribution was concentrated between 18 and 30 years, with 62.8% aged 18–24 and 30.1% aged 25–30, aligning with the typical international student demographic profile. In terms of cultural background, 53.1% of participants were from Asian countries, 22.6% from African countries, 14.2% from European countries, and 10.1% from other regions such as South America and Oceania. This distribution ensured robust cross-cultural variation for empirical investigation. Regarding academic level, 58.4% of respondents were undergraduate students, 29.6% were master's students, and the remaining 12.0% included doctoral candidates and non-degree exchange students. Additionally, more than 90% of participants had resided in China for over 6 months, indicating sufficient cultural immersion for meaningful assessment of adaptation experiences.

**Results:** To further investigate the mechanisms through which CRF influences international students' SWB, this study employed multiple regression and PROCESS mediation analyses based on a sample of 256 international students, using a 7-point Likert scale.

As shown in Table 4, CRF had a significant positive predictive effect on CA (β = 0.550, *p* < 0.001), supporting H1, and significantly predicted SWB (β = 0.494, *p* = 0.018), supporting H2. CRF also significantly negatively predicted IS (β = −0.420, *p* < 0.001), indicating that higher fit perception leads to lower psychological tension.

**Table 4:** Results of study2 regression analysis.

**Path**	**β**	**t**	**SE**	**95% CI**	***p***
CRF SWB	0.494	2.374	0.208	[0.084, 0.904]	0.018^*^
IVO SWB	−0.067	−1.075	0.044	[−0.132, 0.039]	0.283
CRF × IVO → SWB	−0.040	−0.635	0.063	[−0.163, 0.084]	0.528
CRF × IVO → CA	−0.183	−0.238	0.768	[−0.769, 0.133]	0.811
CRF × IVO → IS	0.123	1.5	0.082	[−0.038, 0.286]	0.134
CRF → CA	0.550	14.63	0.038	[0.476, 0.624]	< 0.001^***^
CRF → IS	−0.420	−11.86	0.035	[−0.490, −0.350]	< 0.001^***^
CA → SWB	0.365	6.65	0.055	[0.257, 0.473]	< 0.001^***^
IS → SWB	−0.304	−5.21	0.058	[−0.419, −0.189]	< 0.001^***^
CRF → SWB (direct effect, after mediation)	0.049	0.93	0.052	[−0.054, 0.151]	0.353
**Indirect path**	**Beta**.	**SE**	**95 % Bootstrap CI**	**Whether significant**	
CRF → SWB (Total indirect effect)	0.328	0.042	[0.248, 0.413]	Significant	
CRF → CA → SWB	0.201	0.036	[0.134, 0.275]	Significant	
CRF → IS → SWB	0.128	0.027	[0.079, 0.184]	Significant	

* *p* < 0.05,

*** *p* < 0.001.

Further analyses revealed that CA positively predicted SWB (β = 0.365, *p* < 0.001), while IS negatively predicted SWB (β = −0.304, *p* < 0.001). After introducing CA and IS as mediators, the direct effect of CRF on SWB was no longer significant (β = 0.049*, p* = 0.353), suggesting a full mediation effect.

Bootstrapping (5,000 samples) confirmed the total indirect effect of CRF on SWB was significant [β = 0.328, 95% CI (0.248, 0.413)]:

The mediating effect via CA (CRF → CA → SWB) was significant [β = 0.201, 95% CI (0.134, 0.275)], supporting H3a, H3b and H3c.

The mediating effect via IS (CRF → IS → SWB) was also significant [β = 0.128, 95% CI (0.079, 0.184)], supporting H4a, H4b, and H4c.

In testing the moderating role of IVO, interaction terms were examined:

The interaction effect of CRF × IVO on SWB was not significant (β = −0.040, *p* = 0.528), thus H5a was not supported.

The interaction effect of CRF × IVO on CA was also not significant (β = −0.183, *p* = 0.811), thus H5b was not supported.

The interaction effect of CRF × IVO on IS was marginal and non-significant (β = 0.123, *p* = 0.134), thus H5c was not supported.

Taken together, the results support H1–H4, but not H5a–H5c, indicating that while cultural regulatory fit enhances wellbeing through increasing cultural adaptation and reducing identity strain, the moderating role of individualistic value orientation was not statistically significant in any tested pathway.

### 4.3 Study 3: robustness tests and supplementary analyses

**Objective:** To verify the robustness of the results of the first two studies and further eliminate the influence of common method bias, Study 3 was designed as a time-delay tracking measurement experiment. The SWB scores of the subjects were collected again in the form of time delay to observe whether CRF has a continuous predictive effect on subjective wellbeing. In addition, to further test the cross-cultural applicability of the study model, this study selected a sample of international students from different cultural backgrounds and conducted measurements under the same context Settings to compare the consistency of variable relationships among different subgroups.

**Participants:** In Study 3 (T3 stage), we conducted a delayed follow-up survey 2 months after the completion of T2 to assess the temporal stability and cross-cultural invariance of key relationships. A total of 210 questionnaires were distributed, and 196 valid paired responses were obtained, corresponding to a tracking rate of 86.7% based on the T2 sample. Among these 196 respondents, 47.0% were male and 53.0% were female, with a mean age of 24.3 years (SD = 3.9). In terms of cultural background, 49.5% of the participants were from Asian countries (e.g., Thailand, Vietnam, Pakistan), 27.6% from African countries (e.g., Nigeria, Kenya, Egypt), and 22.9% from Latin America and other regions (e.g., Brazil, Argentina, Oceania). Regarding educational level, 62.2% were undergraduate students, 28.6% were master's students, and 9.2% were either doctoral students or non-degree exchange participants. The T3 questionnaire reassessed SWB as a delayed outcome variable to conduct robustness checks and examine the temporal consistency of the proposed mediation and moderation effects. Additionally, this dataset enabled multi-group analyses to explore the cultural invariance of the model. All respondents demonstrated sufficient language proficiency in either English or Chinese, ensuring the reliability of the data collected.

**Result:** The delay measurement results show that CRF still has a significant positive predictive effect on SWB at time point T1 for time point T2 (β = 0.317, *t* = 3.12, *p* = 0.002), indicating that its influence has a certain degree of persistence. Further introduction of CA as a mediating variable showed that the direct effect of CRF on SWB decreased to insignificant (β = 0.072, *p* = 0.229), while the indirect effect through CA was significant [β = 0.245, Bootstrap 95% CI = (0.161, 0.345)] It once again confirmed the mediating mechanism of CA and was consistent with the conclusion of Study 2.

To further test the applicability of the study model among different cultural background groups, this study used multi-group path analysis, with cultural background (Asian cultural group vs. non-Asian cultural group) as the grouping variable, To examine whether the structural path of the model is structurally invariance between the two groups. The analysis showed that the χ^2^ differences in most of the model's paths were not significant, indicating that the model had good cross-cultural stability across different cultural groups.

As shown in Table 5 The influence of CRF on CA (Δχ^2^ = 2.15, *p* = 0.143), on IS (Δχ^2^ = 2.91, *p* = 0.088), and its direct effect on SWB (Δχ^2^ = 1.05). There were no significant differences between the groups. Meanwhile, the negative path of IS on SWB (Δχ^2^ = 1.58, *p* = 0.209) also shows invariance. These results suggest that the effect of CRF as a moderating mechanism on the SWB pathway is generally robust across different cultural contexts.

**Table 5:** Multi-group path analysis with cultural background as grouping variable.

**Path relationship**	**Free model χ^2^ (df)**	**Constrained model χ^2^ (df)**	***Δχ*^2^ (df)**	***p***	**Whether significant**
CRF → CA	1242.36 (806)	1244.51 (807)	2.15 (1)	0.143	Not significant
CRF → IS	1242.36 (806)	1245.27 (807)	2.91 (1)	0.088	Not significant
CA → SWB	1242.36 (806)	1247.86 (807)	5.50 (1)	0.019	Significant
IS → SWB	1242.36 (806)	1243.94 (807)	1.58 (1)	0.209	Not significant
CRF → SWB (direct effect)	1242.36 (806)	1243.41 (807)	1.05 (1)	0.305	Not significant

Δχ^2^ values are calculated as the χ^2^ of the constrained model minus the χ^2^ of the free model; all df differences = 1; p-values are estimated using the χ^2^ distribution.

However, there was a significant difference in the positive path of CA on SWB between the two groups (Δχ^2^ = 5.50, *p* = 0.019), suggesting that the influence of CA on SWB is moderated by cultural background. Specifically, the standardized regression coefficient of this path was slightly higher in the Asian cultural group than in the non-Asian cultural group, suggesting that CA had a stronger promoting effect on SWB in the group closer to the host culture. This finding suggests that when promoting wellbeing among international students in China, differentiated adaptation support strategies should be provided in light of cultural background differences.

## 5 Discussion

### 5.1 Interpretation of key findings

This study focuses on analyzing the mechanism by which CRF affects the SWB of international students in China. By constructing a dual mediating model that includes CA and IS, and introducing IVO as a moderating variable, it attempts to reveal how fit affects an individual's psychological state and wellbeing level in a real cross-cultural context.

Firstly, the research found that CRF has a significant positive impact on CA. This is in line with the positive effect of cultural support on individual cross-cultural adaptation emphasized by existing studies (Ng et al., 2017; Liao et al., 2021). When international students subjectively feel respect and understanding of their original culture in their home culture, they are more likely to show active psychological engagement and positive cultural interaction. Unlike previous approaches that focused on “external resource” interventions (Tavares, 2024), this study emphasizes subjective harmonization at the individual cognitive level. The sense of fit reflects a kind of “cultural context sensitivity,” when individuals believe their own culture can be inclusive, they are more willing to psychologically lower their defenses and establish a new cultural belonging. Secondly, this study further verified the dual-path influence of fit on SWB by simultaneously introducing CA and IS as mediating variables. This mechanism echoes the cultural conflict perspective, which has gained increasing attention in cultural psychology in recent years, that cross-cultural individuals often face internal contradictions in the process of identity adjustment (Nguyen et al., 2023). Research has found that when individuals have a strong perception of CRF, it not only helps to form positive adaptive behaviors but also significantly alleviates identity conflicts, reduces internal consumption of psychological energy, and thereby enhances their overall sense of wellbeing. This point confirms that IS is not an uncontrollable by-product in the process of CA, but rather a psychological state that can be regulated by preceding variables (Sagiv and Schwartz, 2022). However, the moderating effect of IVO was not as significant as expected. Although theoretically individualists may place more emphasis on the fit between the self and the cultural environment (Triandis, 1989), empirical results show that this variable has a weaker moderating effect on the relationship between fit and happiness. This phenomenon may stem from two aspects: one is that the differences in values in the sample are small, resulting in an insignificant moderating effect; The cultural fit itself has a strong predictive power and may dominate the path in the model, thereby masking the marginal effect of the moderating variable. Unlike earlier studies that emphasized the moderation of cultural values (Singelis et al., 1995), the results of this study suggest that in real cultural interaction, actual interaction experience and cultural acceptance may explain the source of individual wellbeing more than value orientation. This provides a theoretical cautionary note for future research to re-examine the boundaries of the value regulation hypothesis. Finally, the analysis results on cross-cultural applicability are also worthy of attention. While the overall path structure shows good consistency between Asian and non-Asian cultural groups (e.g., most paths do not show significant differences), there are certain differences between the two cultural background groups on the impact of CA on SWB path. This result supports the research trend of asymmetry of cultural effects in recent years, that is, the same variable may produce differences in intensity or direction in different cultural contexts (Bierwiaczonek and Kunst, 2021). This suggests that while cultural adaptation models are structurally universal, the significance of specific paths is still influenced by cultural context. Therefore, in actual cross-cultural educational interventions, tailored support strategies should be adopted for individuals with different cultural backgrounds. For example, for international students from highly collectivist cultural backgrounds, emphasis should be placed on guiding collective belonging and identity resources; for highly individualistic groups, more emphasis should be placed on the space for cultural expression and respect for individuality.

### 5.2 Theoretical contributions

This study focuses on the relatively new variable of CRF, delves deeply into its influence path on SWB, and attempts to expand the existing theories in the following three aspects.

First, the research systematically constructed the concept of CRF and clarified its mechanism of action. This concept is different from the one-way identity or cultural distance indicators in traditional cultural adaptation models. Instead, it emphasizes the interactive cognition between the individual and the host culture, belonging to the psychological dimension of relative cultural integration. In recent years, scholars have come to recognize that adaptation is not merely the adjustment of external behavior, but also the subjective perception of the harmony of the cultural environment. This study responds to this theoretical shift and fills the gap in relevant empirical research.

Second, the study, for the first time, started from the dual-channel mechanism to verify the mediating role of CA and IS between CRF and SWB. Compared with previous studies that focused solely on positive adaptation pathways (e.g., Tavares, 2024), this study points out that the process of CA can both enhance SWB and cause identity distress due to cultural conflicts, resulting in negative psychological consequences. This “coexistence of positive and defensive mechanisms” model perspective provides theoretical support for a more comprehensive understanding of cross-cultural psychological responses.

Thirdly, the study supplements the discussion of boundary conditions in cultural psychology regarding the moderating role of IVO. Although a large number of previous literature suggest that value orientations (such as individualism/collectivism) can significantly regulate the CA process (Sagiv and Schwartz, 2022), the results of this study suggest that the moderating effect of intrinsic values may be suppressed in strongly fitting emotional situations. This finding provides an empirical basis for the “context-dependent” nature of value regulation mechanisms and an important reference for future definitions of the scope of action of value dimensions.

### 5.3 Practical contributions

This study provides valuable insights for universities and policy-makers aiming to support the adaptation and wellbeing of international students in China. First, institutions should align their cultural environment with students motivational tendencies. By offering programs that emphasize either achievement and exploration or safety and responsibility, universities can better accommodate diverse student needs and create a more supportive psychological climate. Second, the findings emphasize the importance of reducing identity strain while strengthening cultural integration. Support measures should go beyond academic assistance to include culturally inclusive spaces, mental health services, and peer mentoring programs that help students navigate dual cultural identities and reduce psychological stress. Third, the study underscores that adaptation is a continuous process. Institutions should provide long-term, responsive support that evolves with students over time. Monitoring their adjustment at different academic stages and offering tailored guidance can improve their wellbeing, academic performance, and overall satisfaction, while also strengthening the institution's ability to attract and retain international talent.

### 5.4 Research limitations and future research directions

Although this study has achieved positive results in theoretical construction and empirical testing, several limitations need to be acknowledged. First, despite the use of longitudinal tracking to enhance the dynamic representativeness of the data, this study was not strictly longitudinal in design. Specifically, it did not conduct comprehensive comparisons among different measurement stages nor rigorously examine temporal causality through methods such as Cross-Lagged Panel Modeling (CLPM). Therefore, the causal relationships among cultural regulatory fit, cultural adaptation, identity strain, and subjective wellbeing need to be interpreted with caution. Future research could adopt rigorous longitudinal methodologies and analytical approaches such as CLPM to more precisely validate the temporal dynamics and causal directionality between these variables. Second, the generalizability of the findings might be limited due to sample biases. Because the current sample primarily came from universities located in eastern China, differences related to regional cultural contexts and resource availability could potentially affect the external validity of the conclusions. Future research should broaden sample coverage, including universities in the central and western regions and those situated in non-first-tier cities, to improve the generalizability and applicability of the proposed theoretical model. Third, although IVO was introduced as a moderating variable, empirical results did not support its hypothesized moderating effect. One possible reason could be that the operationalization of IVO was biased toward a macro-level measurement, which might not sufficiently capture subtle individual psychological differences. Future studies could consider introducing more fine-grained psychological variables, such as social comparison tendencies or self-boundary sense, to more accurately uncover the mechanisms underlying the interactions between cultural values and regulatory fit. Finally, despite the overall structural stability of the model across different cultural contexts, some paths varied significantly between groups. This suggests that while the model exhibits a general structural consistency, the underlying mechanisms might vary across different cultural contexts. Future research could further investigate additional culturally sensitive variables, such as complexity of cultural identity or perceived cultural diversity, to deepen understanding regarding how cultural background differences moderate the adaptation process.

## 6 Conclusions

This study explored how CRF influences the SWB of international students in China, proposing a dual mediating mechanism involving CA and IS, with IVO as a moderator. The empirical findings demonstrated that CRF significantly enhances CA and simultaneously reduces IS, both of which play critical mediating roles in improving students‘ SWB. Contrary to theoretical expectations, the moderating effect of IVO was not significant, indicating that direct perceptions of cultural fit might outweigh the impact of inherent value orientations in real cross-cultural contexts. Multi-group analyses confirmed the robustness and structural consistency of the model across Asian and non-Asian student groups, though specific path intensities varied, notably in the CA to SWB link, emphasizing the contextual sensitivity of cultural adaptation processes. This study enriches cultural psychology by uncovering the dual-path (adaptive vs. defensive) cognitive-emotional mechanisms linking CRF and wellbeing. Practically, it highlights the importance of tailored institutional interventions focusing on psychological fit and identity integration to enhance international students' adaptation and overall experience in host countries.

## Data Availability

The raw data supporting the conclusions of this article will be made available by the authors, without undue reservation.

## Appendix

**I. CRF, 6 items**

Refer to Higgins' (2005) theory of moderative focus combined with perception of cultural values.

I feel that my behavioral style matches the cultural values in China.

My thinking style is appropriate in the social contexts of China.

I believe my way of expressing myself is easily understood and accepted in China.

I can naturally adjust my behaviors when interacting with Chinese people.

My lifestyle in China does not conflict much with the local culture.

I find that the value orientation in Chinese culture aligns with my own regulatory orientation.

**II. CA, 6 items**

Reference Ward and Kennedy (1999) Cultural Adaptation Two-dimensional Scale (Behavioral adaptation and emotional adaptation).

I feel I can handle everyday life in China with ease.

I understand the behavioral and communicative patterns of Chinese people.

I can establish positive interpersonal relationships with Chinese friends.

I feel I have emotionally adapted to Chinese culture.

I feel comfortable when interacting with Chinese people.

I have gradually adapted to China's pace of life and social norms.

**III. IS, 6 items**

Refer to the cultural identity conflict Scale of Ward et al. (2011).

I often feel my cultural identity cannot be fully expressed in China.

I am uncertain about which cultural group I belong to.

Sometimes I feel that my role in Chinese society is ambiguous.

Living in China requires me to give up parts of my original identity.

Conflicts between cultural values make me confused.

I feel a sense of inner tension in my life in China.

**IV. SWB, 5 items**

Refer to Seligman (2011) simplified Chinese version of the PERMA model.

I feel that my life has meaning.

I usually feel positive and optimistic about the future.

I feel fulfilled and supported in my interpersonal relationships.

I am satisfied with my accomplishments.

I often experience a state of engagement and fulfillment.

**V. IVO, 6 items**

Refer to the dimensions of “horizontal individualism” and “vertical individualism” in the Individualism/collectivism scale of Sivadas et al. (2008).

Successfully completing a task gives me more satisfaction than working in a team.

I prefer to think and act independently.

I believe everyone should be responsible for their own success.

It is important for me to achieve more than others.

I want others to acknowledge that I am independent and self-directed.

Social status is an important measure of success for me.
